# Lifestyle Medicine in Focus: A Cross-Sectional Study Comparing Domestic and International Students

**DOI:** 10.3390/healthcare12111151

**Published:** 2024-06-06

**Authors:** Eszter Kovács, Viktor Rekenyi, Anna Nánási, Csongor István Szepesi, Putu Ayu Indrayathi, Nóra Horváth, Angéla Csirmaz, Gréta Marjai, Kolozsvári László Róbert

**Affiliations:** 1 Doctoral School of Health Sciences, University of Debrecen, 4032 Debrecen, Hungarynanasi.anna@med.unideb.hu (A.N.); indrayathi.ayu@med.unideb.hu (P.A.I.);; 2 Department of Family and Occupational Medicine, Faculty of Medicine, University of Debrecen, 4032 Debrecen, Hungary; 3 Faculty of Medicine, University of Debrecen, 4032 Debrecen, Hungary

**Keywords:** lifestyle medicine, Hungarian, international, university students, nutrition, physical activity, mental health

## Abstract

This study investigated lifestyle factors among Hungarian and international students, utilizing lifestyle medicine principles to enhance overall well-being. Conducted at the University of Debrecen through a cross-sectional survey, we examined selected lifestyle factors, more specifically sleep patterns, weight management, nutrition, physical activity, mental well-being, and alcohol use. Hungarian (N = 122) and international (N = 139) students were compared, revealing significant differences. Hungarian students slept less (*p* = 0.041), desired weight loss (*p* = 0.040), ate more fruits and vegetables (*p* = 0.014), exercised longer (*p* = 0.002), and reported higher purpose and social support (*p* = 0.009), with higher alcohol use (*p* < 0.001). These findings highlight distinct experiences and challenges faced by these student groups including variations in sleep, weight management, diet, exercise, and social support. Targeted interventions and tailored support are essential to address their specific needs. To promote the well-being of both Hungarian and international students, educational programs addressing various facets of a healthy lifestyle are crucial. This study offers valuable insights into lifestyle factors and health outcomes among Hungarian and international students and underscores the importance of addressing the unique needs of each group through tailored interventions.

## 1. Introduction

The lifestyle of international students is a topic of growing interest and importance in academia and public health. As students move to foreign countries for educational pursuits, they face numerous challenges and adjustments that can significantly impact their well-being [1,2]. Understanding the unique aspects of their lifestyle including diet, physical activity, sleep patterns, and psychosocial factors is crucial for promoting their health and academic success [2].

The significance of lifestyle started to increase in the second half of the 20th century due to the changing nature of prevalent diseases. Previously, the most common diseases were typically acute and predominantly infectious. However, these have increasingly been replaced by chronic diseases. Lifestyle change as a frontline treatment has been present in clinical practice for several decades. However, lifestyle medicine is a relatively new, scientifically grounded medical specialty [3].

Lifestyle medicine is primarily a preventive approach that focuses on preventing diseases (primary prevention), but also holds important therapeutic significance. Lifestyle medicine applies various lifestyle interventions in the treatment of chronic diseases including obesity, diabetes, and cardiovascular diseases. It has six pillars, predominantly plant-based nutrition, regular physical activity, adequate quantity and quality of sleep, stress management, avoidance of harmful addictions, and nurturing social connections [4].

### 1.1. Healthy Diet

Healthy nutrition is essential for maintaining overall health and preventing chronic diseases. A balanced diet includes a variety of nutrients from different food groups, emphasizing fruits, vegetables, whole grains, lean proteins, and healthy fats. Consuming a diverse range of foods ensures that the body obtains the necessary vitamins, minerals, fiber, and antioxidants. Key dietary patterns have been extensively studied for their health benefits.

Fruits and vegetables are critical components of a healthy diet. They are rich in vitamins, minerals, fiber, and antioxidants, which are vital for preventing chronic diseases. The World Health Organization (WHO) recommends consuming at least 400 g of fruits and vegetables per day to reduce the risk of heart disease, stroke, and certain cancers [5]. Epidemiological studies have consistently shown that a higher intake of fruits and vegetables is associated with a lower risk of all-cause mortality [6].

Whole grains including brown rice, oats, quinoa, and whole wheat products are excellent sources of complex carbohydrates, fiber, and essential nutrients. Replacing refined grains with whole grains can lower the risk of cardiovascular diseases, type 2 diabetes, and obesity. A meta-analysis found that a higher consumption of whole grains is inversely associated with the risk of chronic diseases and mortality [7].

Lean proteins are vital for the growth, repair, and maintenance of tissues. Sources include poultry, fish, beans, lentils, and low-fat dairy products. Fish, in particular, provides omega-3 fatty acids, which are beneficial for heart health. The Dietary Guidelines for Americans recommend incorporating a variety of protein sources and limiting red and processed meats to reduce the risk of cancer and cardiovascular diseases [8].

Healthy fats such as those found in olive oil, avocados, nuts, and seeds are important for reducing bad cholesterol levels and supporting heart health. Unsaturated fats should replace trans fats and saturated fats, which are linked to an increased risk of cardiovascular disease. The American Heart Association advises choosing unsaturated fats to promote heart health [9].

Dietary fiber, abundant in fruits, vegetables, whole grains, and legumes, is crucial for digestive health. It helps maintain bowel regularity, lowers cholesterol levels, and supports a healthy weight. High fiber intake is associated with a reduced risk of various conditions including heart disease, stroke, hypertension, diabetes, and certain gastrointestinal diseases [10].

### 1.2. Physical Activity

The importance of regular physical activity is widely recognized today. It has been proven to reduce the risk of all-cause mortality, high blood pressure, stroke, coronary heart disease, metabolic syndrome, type 2 diabetes, and the development of colorectal and breast cancer [11,12]. Additionally, it improves cognitive function and fitness levels, and has positive effects on bone health [11,12,13].

Researchers have investigated the factors underlying the positive effects of physical activity. It appears that one of them is its anti-inflammatory effect, as exercise can reduce chronic low-grade inflammation [14,15]. Sedentary individuals tend to have higher amounts of adipose tissue and excess weight. The bodies of overweight and especially obese individuals are in a constant state of low-grade inflammation, which is particularly unfavorable for our health [13].

The World Health Organization (WHO) recommends that adults (aged 18–64) engage in at least 150–300 min of moderate-intensity aerobic physical activity per week, or at least 75 min of vigorous-intensity aerobic activity per week. Additionally, it is important to incorporate muscle-strengthening activities involving major muscle groups at least twice a week [16]. The relationship between mental health and exercise was examined in a recent study. The study compared the effectiveness of antidepressants (citalopram, sertraline) and running therapy in patients with depression or anxiety disorders. Some of the patients received antidepressant treatment (45 participants), while others participated in group running sessions (96 participants) at least twice a week. At the end of the 16-week study period, the remission rates were similar in both groups. In addition to the positive mental effects, the participants’ physical condition also improved [17].

International students face several barriers when it comes to engaging in physical activities. Language can be challenging, as understanding instructions or communicating with fitness professionals may be difficult. The feeling of isolation, being away from familiar social support networks, can discourage participation [18]. Financial constraints and increased costs associated with gym memberships or sports equipment can also pose barriers. Additionally, the demanding academic schedule and commitments may leave international students with limited time to allocate to physical activities [19,20]. Lastly, religious beliefs and practices may influence the types of activities international students feel comfortable participating in. These barriers highlight the importance of creating inclusive and supportive environments that address the unique needs of international students to promote their engagement in physical activities [19,20,21].

### 1.3. Sleep

According to the recommendations of the American Academy of Sleep Medicine (AASM), adults should aim for a minimum of 7 h of sleep for optimal health. Individuals who consistently sleep less than this recommended amount are at increased risk of certain adverse health outcomes. Similarly, regular sleep durations exceeding 9 h are also not ideal, except in certain exceptions such as children, young adults, individuals who are ill, or those who are sleep-deprived [22].

Sleep disorders are frequently observed among students, particularly international students, who face the challenges of adapting to a new environment in a foreign country [23]. The stress experienced by international students (ISs) due to various factors also impacts their sleep patterns. In many cases, ISs suffer from sleep deprivation, which can have negative effects on their academic performance [24]. In a study conducted in China, it was found that domestic students had a longer sleep duration (over 6 h) compared to international students, and a shorter sleep duration was associated with a higher percentage of body fat and a higher body mass index [25].

### 1.4. Stress Management

The increased awareness of the impact of unhealthy lifestyles on the emergence and persistence of anxiety symptoms has generated curiosity regarding the utilization of lifestyle medicine as a potential strategy for managing anxiety [26].

While stressors are widely recognized as significant factors in the development and progression of chronic diseases, public health recommendations often emphasize stress management less than diet and exercise [27]. However, the capacity of individuals to modify their behavior and response to stressors plays a vital role in personalized lifestyle medicine. Without addressing behavioral adjustments and the locus of control concerning stressors, it can be challenging to implement dietary and activity changes effectively. Therefore, it is essential to consider the fundamental behavioral aspects of lifestyle medicine to strengthen the necessary physical transformations for improved health [27,28].

When international students embark on the journey of pursuing an academic degree in a foreign country, they encounter a mix of excitement and challenges. They may face various adjustment issues that can significantly impact their overall experience throughout their studies. These challenges include culture shock, discrimination, financial constraints, social isolation, proficiency in the English language, academic difficulties, unmet expectations, employment concerns, and psychological distress [29].

### 1.5. Harmful Addictions

Based on the Hungarian Central Statistical Office (KSH) data in 2019, 27.7% of men and 22.3% of women in Hungary smoke cigarettes daily, while 4.7% smoke occasionally. Additionally, 36.4% of men and women have successfully quit their harmful habits [30]. Regarding alcohol consumption, according to KSH data in 2019, 9.3% of Hungarian men identified themselves as heavy drinkers, while 30.9% considered themselves moderate drinkers. The percentages were lower for women, with 1.5% being heavy drinkers and 10.6% being moderate drinkers [31].

Individuals among international university students experiencing higher levels of acculturative stress were found to be more vulnerable to addictive behaviors including smoking and alcohol consumption, as indicated by studies [1,32]. Another significant consequence of acculturative stress is reduced resilience, which was identified as a mediating factor in the relationship between stress levels and binge drinking, as observed in one study [33].

### 1.6. Life Satisfaction and Social Connections

The lack of quality relationships has a negative impact on our mental and physical health. In 2010, researchers conducted a meta-analysis, reviewing 145 prospective studies on the subject. They found that individuals who reported having closer relationships had a 50% higher likelihood of survival compared to those with less close relationships [34]. The existence of this association was already suggested by a prospective study conducted in 1988. The results showed that individuals with fewer social connections tended to have a shorter lifespan compared to their socially active peers [35]. The relationship between loneliness, depression, and cognitive functions was examined in an older American population. The study included over 8000 participants who were aged 65 and above. The study took place between 1998 and 2010, and the participants were reassessed every two years, focusing on loneliness, depression, cognition, health status, and social and demographic factors. The results indicated that initially, lonely individuals experienced accelerated cognitive decline during the study period, regardless of depression, health status, social network, and societal and demographic factors [36].

The presence of social connections plays a crucial role in the socio-cultural adjustment of international students during the process of acculturation [37]. However, various factors can significantly hinder the international student’s ability to form healthy social relationships. Experiences of perceived discrimination, social exclusion, and feelings of loneliness are associated with higher levels of acculturative stress among international students [38,39]. A study conducted in Hungary during the COVID-19 pandemic showed that international students had significantly decreased social support compared to domestic students. Female international students showed more depressive symptoms even after the lifting of pandemic regulations [40].

Recognizing the crucial significance of life satisfaction, one study underscored that the well-being of international students studying in China hinged significantly on various factors. Enhancing the satisfaction levels of international students entails focusing on enhancing four key areas: the quality of the offered courses, increased utilization of the English language for communication across all levels, the development of support services, and the expansion of counseling services [41].

A study in Ghana revealed that psychosocial factors like friendship count, financial satisfaction, and perceived discrimination significantly influenced psychological adaptation, each impacting the outcomes differently. While perceived discrimination affected both adaptation indicators significantly, the others impacted only one outcome significantly. Surprisingly, English proficiency worsened psychological symptoms, suggesting that higher proficiency correlated with more psychological distress [42].

Based on this view, our study aimed to compare the lifestyle patterns and behaviors of international and domestic Hungarian students to identify any differences and understand the unique challenges international students face in relation to their health and well-being.

## 2. Materials and Methods

Data collection started in September 2021 at the Occupational Health Service of the University of Debrecen. The participants of our study were primarily international students who arrived for their occupational examination. A total of 145 individuals completed the questionnaire here, and data collection was voluntary and anonymous. The second phase of data acquisition took place in November 2021 during family medicine seminars attended by fifth-year Hungarian medical students. Data collection was also conducted voluntarily, and 124 participants completed the anonymous questionnaire. The questionnaires were presented in the English language for both domestic and international students. The exclusion criteria for our study comprised individuals who were not university students, did not speak English fluently, were under the age of 18, and those who had not completed the questionnaires. After excluding six international students and two Hungarian students, the total number of data was 261. The validation process was not executed because of the limited participant pool and the convenient sampling method used. However, an assessment of internal consistency was conducted using Cronbach’s alpha.

### 2.1. The Questionnaire

The questionnaire used in this study was jointly developed by the American College of Lifestyle Medicine (ACLM) and Loma Linda University [43]. No similar questionnaire has been developed in the Hungarian language on this topic. In addition to the ACLM questionnaire (Lifestyle Assessment Short Form), sociodemographic and anthropometric data were also collected.

In the questionnaire, participants were asked to assess their current health status on a scale of 0–10, where 0 represented very poor health and 10 represented excellent health. Following this, the respondents provided answers to questions related to their sleep habits and indicated the statements that characterized their experiences over the past two weeks. Participants were asked to specify the number of hours they slept, choosing from the following options: (a) less than 4 h, (b) 4–5 h, (c) 6 h, (d) 7–8 h, (e) 9 or more hours. They also reported how often they felt tired or had difficulty staying awake during their daily routine tasks in the past two weeks.

The next section focused on the participants’ satisfaction with their current weight. Five options were provided: (a) I want to gain a lot of weight, (b) I want to gain a little weight, (c) I am satisfied with my weight, (d) I want to lose a little weight, (e) I want to lose a lot of weight. Regarding nutrition, participants were asked to indicate the frequency of consuming fast food, sugary drinks, and processed foods in the past two weeks. The response options were: (a) not at all, (b) on multiple days, (c) on more than half of the days, (d) nearly every day. Participants were then asked to report the average number of servings of fruits and vegetables they consumed on a typical day (one handful of fruit or vegetables counted as one serving). The response options for this question were: (a) less than 2 servings, (b) 2–3 servings, (c) 4–5 servings, (d) more than 5 servings.

Students were also required to provide information about their physical activity habits. They were asked to indicate the frequency of engaging in moderate or strenuous intensity physical activity during the past two weeks. Participants could choose from the following options: (a) less than 1 time per week, (b) 1–2 times per week, (c) 3–4 times per week, (d) 5 or more times per week. For each physical activity occasion, participants were asked to report the duration of the activity, ranging from (a) less than 10 min, (b) 10–29 min, (c) 30–49 min, (d) 50 or more minutes.

In the section of mental health, the questions also focused on experiences over the past two weeks including feelings of decreased interest, decreased pleasure, depressive mood, feelings of hopelessness, and sense of belongingness to a community. Students were asked to rate these experiences using a 4-point Likert scale, where 0 represented the absence of such experiences.

The questionnaire also included questions about substance use, specifically nicotine, alcohol, designer drugs, and marijuana. Participants were asked whether they had ever used the respective substance, the frequency and quantity of use, and the extent to which they felt concerned about substance use (on a scale of 0–5, where 0 indicated no concern and 5 indicated high concern).

The questionnaire concluded with questions about personal motivation. Participants were asked to select three categories from the given options that they felt most motivated to change. These categories aligned with the pillars of lifestyle medicine. Lastly, the participants were asked to provide a written statement describing what motivates them to adopt a healthier lifestyle.

Due to the low number of participants indicating drug and marijuana use, and insufficient responses regarding personal motivation, these aspects were excluded from the study.

### 2.2. Data Analysis

For the statistical analysis, IBM SPSS Statistics version 26.0 was utilized and a significance level of *p* < 0.05 was considered statistically significant. Descriptive statistics were computed to summarize the participants’ sociodemographic characteristics including gender, faculty, marital status, BMI, and age. Categorical variables were presented as frequencies, while continuous variables were reported as medians due to non-normality (to test the sample distribution, we used the Kolmogorov–Smirnov and Shapiro–Wilk tests). To examine the differences between the Hungarian and international students, Fisher’s exact test or Chi-square tests were used depending on cell frequency. To compare medians, we utilized the Mann–Whitney U-test. Spearman rank correlation was used to investigate the correlation between body mass index (BMI) and self-assessed overall health. Furthermore, stepwise regression was utilized in the analysis to systematically select the most relevant independent variables that contributed significantly to predicting the dependent variable. Using stepwise regression, we aimed to identify the most influential factors among the questionnaire items and demographic data associated with overall health.

### 2.3. Ethical Consideration

We obtained ethical approval for the use of questionnaires and in conducting our research on 15 December 2021, from the Regional and Institutional Research Ethics Committee of the Clinical Center of the University of Debrecen. The reference number is 5935-2021.

## 3. Results

Cronbach’s alpha was calculated to measure the reliability of the questionnaire items. The Cronbach’s alpha coefficient was found to be 0.297, indicating low internal consistency.

The sociodemographic characteristics presented in Table 1 show that in terms of gender, the majority of participants identified as female and only one of the Hungarian students chose the other option, identifying as non-binary. When examining the distribution according to university faculties, we found that most of the participants belonged to healthcare-related faculties. Regarding marital status, single students dominated the sample. International students were significantly younger and had higher BMI values than domestic students.

### 3.1. Differences in Lifestyle among Hungarian and International Students

Table 2 shows the frequency distributions and chi-square test outcomes for various lifestyle factors among the Hungarian and international students. The results revealed significant differences between the two groups in terms of sleep duration, weight perception, fruit and vegetable consumption, exercise duration, mental well-being, and alcohol use.

#### 3.1.1. Sleep

Hungarian students slept more compared to international students, but according to tiredness, there was no significant difference.

#### 3.1.2. Weight Management

Most of the students wanted to lose weight in both samples. The international students tended to be happier with their weight or wanted to gain weight compared to the Hungarian participants.

#### 3.1.3. Nutrition

No significant difference was found in the consumption of fast food products between the students. In both samples, most of the students ate 2–3 servings of healthy food on an average day, and Hungarian students were more likely to eat more servings of healthy food.

#### 3.1.4. Exercise

In terms of days over the last two weeks spent with exercise at a moderate strenuous intensity, there was no significant difference between the participants, but in an average session, the Hungarian students tended to do more exercise.

#### 3.1.5. Purpose and Connection/Mental Health

Over the last 2 weeks, students in the Hungarian sample felt like their life had a purpose or meaning and looked for support more frequently. There was no significant difference between the samples in terms of being bothered by little interest or pleasure in doing things, feeling down, depressed, hopeless, nervous, anxious, or worrying too much.

#### 3.1.6. Smoking/Substance Use

We investigated the differences in alcohol and nicotine abuse. Only alcohol consumption showed significant differences, showing that there were more students in the international sample who had not used alcohol in the past year.

#### 3.1.7. Overall Health

There was no significant correlation (*p* = 0.594) in terms of self-assessed overall health between the Hungarian (M = 8.00) and international students (M = 8.00), however, BMI showed a significant weak negative correlation with this factor in the overall sample (r = −0.132, *p* = 0.034).

### 3.2. Predictors of Overall Health

To ensure clarity and enhance the reliability of our analysis, we made certain exclusions from the independent variables. Specifically, we excluded marijuana and recreational drug usage due to the limited number of participants who reported using them. Similarly, the scales of alcohol and nicotine dependency were removed to focus on variables with more substantial representation in our study. Additionally, we decided to exclude the “other” category from the gender variable as it was chosen by only one participant, which could potentially introduce bias. Furthermore, motivational factors were excluded due to the small proportion of responses received in this category.

The results of the stepwise multiple linear regression analysis revealed several predictors significantly associated with overall health. Being married or in a relationship demonstrated a positive correlation, while factors like worrying too much over the last 2 weeks, identifying as male, and wanting to lose weight were in negative correlation with overall health (Table 3).

## 4. Discussion

Our study examined various lifestyle factors among Hungarian and international students and identified significant differences between the two groups in the aspects of lifestyle medicine. These findings provide valuable insights into the unique challenges and experiences faced by these student populations.

Cronbach’s alpha was calculated to measure the reliability of the questionnaire items. The resulting coefficient indicated low internal consistency, suggesting that the items in the questionnaire may not be reliably measuring the same underlying construct. This highlights the need for further refinement and validation of the questionnaire.

Addressing the potential contradiction raised, while there were several differences observed in healthy habits among the Hungarian and International students, it is noteworthy that the self-assessed overall health did not significantly differ between the two groups. This discrepancy underscores the complex nature of health perceptions and the multifaceted factors that contribute to individuals’ assessments of their own health status. Thus, while lifestyle behaviors may differ, the subjective perception of overall health remained relatively consistent across the Hungarian and International student populations.

BMI showed a significant weak negative correlation with overall health, indicating that higher BMI values were associated with lower perceived health. This finding aligns with previous research highlighting the negative impact of higher BMI on various health outcomes [44] and underscores the importance of promoting healthy lifestyle choices including maintaining a healthy weight to support the overall well-being of students.

Sleep patterns also differed between the Hungarian and international students. Hungarian students reported sleeping more on average over the last two weeks compared to the international students. However, there was no significant difference in terms of tiredness. These findings may reflect cultural and individual variations in sleep habits as well as the impact of academic and social demands on sleep duration [23]. A study revealed that poor sleep and sleep disturbances were prevalent among long-staying international graduate students in Japan, however, many did not seek professional assistance for these issues. Thus, disseminating sleep hygiene information among this demographic to enhance sleep quality is deemed essential [45]. Promoting healthy sleep habits and raising awareness about the importance of quality sleep for overall well-being and academic performance is crucial for both groups of students.

Weight management was an area of concern for both Hungarian and international students, with a significant proportion of students expressing a desire to lose weight. It was interesting to note that the international students were more satisfied with their current weight or even wanted to gain weight, despite having a higher BMI compared to the Hungarian students. This suggests that cultural factors and individual perceptions of weight play a role in these differences [46]. However, both of their BMI values were in the normal range. Our study findings regarding the relationship between BMI values and overall health are aligned with previous research highlighting the negative impact of higher BMI on perceived overall health. Several studies have demonstrated a significant association between higher BMI and lower self-rated health [47,48].

In terms of nutrition habits, there was no significant difference in the consumption of fast food products between the Hungarian and international students. Hungarian students were more likely to eat more servings of healthy food. International students, on the other hand, faced various barriers when it came to maintaining healthy eating habits. These barriers included the high cost of traditional foods compared to fast food options, the poor quality of regular food choices, lack of time and knowledge required for food preparation, challenges related to food preparation methods, taste, appearance, and neophobia, which is the fear of trying new foods [49,50,51]. Additionally, religious orientations can affect their access to specific ethnic cuisines such as Arabic, Japanese, or Chinese food. The concept of nutrition in the host country and the available time for food-related activities further contribute to the difficulties faced by international students in establishing and maintaining a nutritious diet [49].

Exercise frequency did not significantly differ between the Hungarian and international students. Domestic students tended to spend more time exercising per session on average. These findings indicate that both groups engage in physical activity, but the duration of exercise sessions may vary. Despite the well-documented evidence highlighting the positive health benefits of physical activity, international students might exhibit lower participation rates in physical activity due to the influence of acculturative processes [52] and sociocultural factors associated with their communities of origin [53,54].

Regarding mental well-being, Hungarian students reported feeling like their life had purpose or meaning and looked for support over the last two weeks more frequently compared to the international students. This indicates that international students may face challenges in finding adequate social support networks and resources to help them navigate their college or university experiences as they are far away from their significant resources of support [40]. However, it is noteworthy that there was no significant difference between the samples in terms of other indicators of mental well-being such as feelings of depression, hopelessness, or excessive worrying. This indicates that while there may be disparities in certain aspects of mental well-being between Hungarian and international students, other factors related to mood and emotional state appeared to be consistent across the two groups.

Alcohol consumption in the last year was higher among domestic students, which also indicates cultural differences. Alcohol consumption is a global phenomenon, but its perception and cultural significance vary across different societies. In certain cultures, alcohol holds a significant role in communication and business interactions. Conversely, other cultures promote the idea of moderate alcohol consumption or even complete abstinence [55].

Consistent with our findings, studies have reported that being married or in a committed relationship is linked to better health outcomes [56,57]. Furthermore, the negative correlation between excessive worrying and overall health is supported by evidence demonstrating the detrimental effects of stress on physical and mental well-being [58]. The negative impact of weight-related concerns on overall health is further underscored by research indicating the adverse effects of body dissatisfaction and weight stigma on psychological and physical well-being [59]. When considering the broader context of lifestyle factors, it becomes evident that these issues play a significant role. Additionally, male students may perceive their overall health as poorer due to their engagement in health-risk behaviors and their tendency to underutilize healthcare services [60]. This highlights how these factors intertwine with and influence lifestyle choices and habits, ultimately affecting one’s well-being

### Limitations

The study had several limitations that should be acknowledged. First, the sample used in the study may not be representative of the broader student population as it primarily consisted of medical students in the Hungarian sample. It is important to note that both samples comprised students from the University of Debrecen. Additionally, low participant responses regarding drug and marijuana use as well as personal motivation restricted the inclusion of these aspects in the analysis. It is also worth mentioning that the ACLM’s Lifestyle Assessment Short Form, although widely used, has not been specifically validated for the Hungarian population. Another limitation of our study includes the lack of examination of specific cultural differences between Hungarian and international students. While we acknowledged the presence of international students, we did not have a sufficient number of students from each country to analyze them separately. This limited our ability to explore potential country-specific factors that may influence lifestyle choices and health outcomes. Another limitation of this study is the questionnaire’s low internal consistency, indicating that further improvement is needed in its design and formulation. This aspect could have influenced the reliability of the data collected and potentially affected the accuracy of the results. Future research with larger and more diverse samples is needed to address these limitations.

## 5. Conclusions

Our study provides important insights into the lifestyle factors and health outcomes among Hungarian and international students. The findings highlight the need for targeted interventions and support systems to address the unique challenges faced by international students. Furthermore, our discussion highlighted the significance of other factors such as sleep patterns, weight management, nutrition habits, exercise frequency, and mental well-being in shaping the overall health and well-being of students. Addressing these factors comprehensively through education programs focusing on various aspects of a healthy lifestyle is crucial. Additionally, emphasizing the importance of personalized lifestyle medicine can empower students to make informed choices aligned with their personal goals and values. Recognizing the individual needs, preferences, and circumstances of students is essential for promoting sustainable behavior change and enhancing their overall well-being. The observation that international students may be less likely to seek social support emphasizes the importance of proactive measures in establishing accessible and culturally sensitive support systems. Additionally, the finding that Hungarian students reported a stronger sense of life purpose underscores the need for interventions aimed at enhancing the sense of purpose and belonging among international students, which can contribute to their overall well-being and academic success. This highlights the significance of creating inclusive environments within educational institutions that address the diverse social and emotional needs of all students, irrespective of their cultural backgrounds or nationalities. In order to promote the well-being of both Hungarian and international students, it is crucial to implement education programs that focus on various aspects of a healthy lifestyle. By tailoring interventions and recommendations to the specific needs of students, personalized lifestyle medicine can empower them to make choices that align with their personal goals and values.

## Figures and Tables

**Table 1:** Sociodemographic characteristics of the participants.

Variable	Hungarian N = 122	International N = 139	Total N = 261	Fisher’s Exact Test/Mann–Whitney U Test
**Gender**				
Female	65	71	136	*p* = 0.616
Male	56	68	125	
Other	1	0	1	
**Faculty**				
Healthcare-related	118	86	204	*p* < 0.001
Not healthcare-related	4	53	57	
**Marital status**				
Single	48	112	160	*p* < 0.001
In a relationship	69	27	96	
Married	4	0	4	
Divorced	1	0	1	
**BMI (median)**	18.71	22.27		*p* < 0.001
**Age (median)**	24.00	22.00		*p* < 0.001

**Table 2:** Frequency distributions and chi-square outcomes of lifestyle factors among the participants.

	Hungarian	International	Total	Chi-Square
**Average sleeping hours over the last two weeks in a 24-h period**				
<7–8 h	38	60	98	χ2(1) = 4.192 *p* = 0.041
7–8 h or more	84	78	162	
**Opinion about current weight**				
Wants to gain weight	15	34	49	χ2(2) = 6.460 *p* = 0.040
Happy with current weight	41	43	84	
Wants to lose weight	66	62	128	
**Servings of fruits and vegetables on an average day**				
Less than 2 servings	30	56	86	χ2(2) = 8.481 *p* = 0.014
2–3 servings	69	68	137	
4–5 servings or more	23	15	38	
**Average minutes spent with exercise during a session**				
10–29 min or less	42	74	98	χ2(1) = 9.312 *p* = 0.002
30–49 min or more	80	65	162	
**Felt like life had a purpose or meaning over the last 2 weeks**				
Not at all or several days	28	56	84	χ2(2) = 9.504 *p* = 0.009
More than half the days	38	29	67	
Nearly every day	55	53	108	
**Connected with any support network over the last 2 weeks**				
Not at all or several days	28	56	84	χ2(2) = 9.504 *p* = 0.009
More than half the days	38	29	67	
Nearly every day	55	53	108	
**Use of alcohol in the past year**				
Yes	89	35	124	χ2(1) = 59.457 *p* < 0.001
No	33	104	137	

**Table 3:** Results of the stepwise multiple linear regression analysis.

Variable	Beta	SE	95% CI	Significance
Marital status	0.239	0.151	0.173; 0.771	0.002
Gender	−0.206	0.151	−0.110; −0.204	0.008
Worrying	−0.190	0.085	−0.044; −0.166	0.014
Wanting to lose weight	−0.156	0.097	−0.006; −0.099	0.043
Adjusted R^2^ = 0.124 F value = 4.166 *p* = 0.043 Dependent value: Overall health				

## Data Availability

The data presented in this study are available on request from the corresponding author. The data are not publicly available due to ethical reasons.
